# Identification of the HDL-ApoCIII to VLDL-ApoCIII ratio as a predictor of coronary artery disease in the general population: The Chin-Shan Community Cardiovascular Cohort (CCCC) study in Taiwan

**DOI:** 10.1186/1476-511X-11-162

**Published:** 2012-11-23

**Authors:** Po-Yuan Chang, Chii-Ming Lee, Hsiu-Ching Hsu, Hung-Ju Lin, Kuo-Liong Chien, Ming-Fong Chen, Chu-Huang Chen, Yuan-Teh Lee, Chao-Yuh Yang

**Affiliations:** 1 Department of Internal Medicine, National Taiwan University Hospital and National Taiwan University College of Medicine, No. 7, Chung-Shan South Road, Taipei, 100, Taiwan; 2 Department of Medicine, Baylor College of Medicine, Houston, Texas, USA; 3 Vascular and Medicinal Research, Texas Heart Institute, Houston, Texas, USA; 4 Graduate Institute of Clinical Medical Science, China Medical University, Taichung, Taiwan; 5 L5 Research Center, China Medical University Hospital, Taichung, Taiwan; 6 Department of Internal Medicine, China Medical University, Taichung, Taiwan

**Keywords:** Apolipoproteins, Coronary artery disease, Lipoproteins, Cardiovascular risk factors, Chin-Shan Community Cardiovascular Cohort (CCCC) Study, High-density lipoprotein (HDL), Very-low-density lipoprotein (VLDL), Apolipoprotein CIII (ApoCIII)

## Abstract

**Background:**

Apolipoprotein (Apo) levels are considered more reliable than plasma lipoprotein levels for predicting coronary artery disease (CAD). However, a unanimous Apo marker for CAD has not been identified. In the Chin-Shan Community Cardiovascular Cohort (CCCC), we sought to identify a common Apo marker for predicting CAD in the general population.

**Methods:**

We examined the cross-sectional association between Apo markers and CAD in the CCCC from 1990 to 2001. Among 3,602 subjects, 90 had angiographically proven CAD (>50% stenosis in ≥1 vessel), and 200 did not have CAD. These subjects were divided into the following 4 groups for analysis: normolipidemic (total cholesterol [TC] <200 mg/dL, triglyceride [TG] <150 mg/dL), hypertriglyceridemic (TC <200 mg/dL, TG ≥150 mg/dL), hypercholesterolemic (TC ≥200 mg/dL, TG <150 mg/dL), and hyperlipidemic (TC ≥200 mg/dL, TG ≥150 mg/dL).

**Results:**

Compatible with findings in other populations, our results showed that CAD patients in the CCCC had higher ApoB and lower high-density lipoprotein (HDL) cholesterol and ApoAI concentrations than non-CAD subjects, but the differences were not significant in all groups. Plasma concentrations of ApoE and lipoprotein (a) were not consistently correlated with CAD. In contrast, the ratio of HDL-ApoCIII to very-low-density lipoprotein (VLDL)-ApoCIII was the only universal determinant for CAD in the normolipidemic group (*P*=0.0018), the hypertriglyceridemic group (*P*=0.0001), the hypercholesterolemic group (*P*=0.0001), and the hyperlipidemic group (*P*=0.0001). Overall, a high HDL-ApoCIII/VLDL-ApoCIII ratio was observed in all CAD patients, including those with a normal lipid profile. In multivariate analyses, the HDL-ApoCIII/VLDL-ApoCIII ratio was the strongest predictor for CAD among all lipid factors investigated (odds ratio, 2.04; 95% confidence interval, 1.46–2.84; *P*<0.0001).

**Conclusions:**

A high HDL-ApoCIII to VLDL-ApoCIII ratio is a better marker for predicting CAD than are the conventional lipid markers or ApoAI and ApoB. High HDL-ApoCIII and low VLDL-ApoCIII values in CAD, irrespective of lipid variations, suggest that ApoCIII is markedly transported from VLDL to HDL in this disease. Measurement of plasma ApoCIII may improve CAD prediction in the general population.

## Background

Dyslipidemia, which includes various lipid and lipoprotein abnormalities, is closely related to coronary artery disease (CAD) [1]. Conventional lipid markers that are considered predictors for CAD include high levels of total cholesterol (TC), triglyceride (TG), and low-density lipoprotein cholesterol (LDL-C) and low levels of high-density lipoprotein cholesterol (HDL-C) [2]. However, a growing body of evidence suggests that altered apolipoprotein (Apo) levels may more accurately predict CAD [3].

Clinical and pathobiologic studies including the Apolipoprotein-related Mortality Risk study have shown that a high ApoB/ApoAI ratio is related to increased incidence of myocardial infarction and stroke [4,5]. Recent studies have shown the enhanced atherogenicity of ApoB lipoproteins and HDL in association with ApoCIII [6-8]. Because ApoCIII is a major component of TG-rich lipoprotein, it delays the catabolism of very-low-density lipoprotein (VLDL) and chylomicrons, resulting in elevated plasma TG concentrations [9]. Studies in transgenic mice have shown that ApoCIII levels in VLDL and HDL are exchangeable and can be modulated by ApoAV to sustain HDL homeostasis [10]. The mechanism linking ApoCIII to CAD may be attributed to the transfer of ApoCIII to HDL during lipolysis [11,12] and to the ability of ApoCIII to enhance monocyte–endothelial cell adhesion [6]. These processes result in a marked change in HDL concentration, which is further complicated by the ability of ApoCIII to inhibit lipolysis and of ApoE to enhance lipoprotein clearance [13,14].

To date, an Apo marker that is consistently associated with CAD in the general population has not been described. To identify such a marker and risk factors for CAD, we performed a cross-sectional study in the community-based Chin-Shan Community Cardiovascular Cohort (CCCC) and analyzed the correlation between CAD and various lipid and Apo abnormalities.

## Materials and methods

### Study design and selection of study subjects

All patients provided informed consent, and the study protocol conforms to the Declaration of Helsinki guidelines. The institutional review board of the National Taiwan University Hospital approved this study [15]. This is a CCCC substudy; details of the CCCC study have been described previously [15]. Briefly, 3,602 adults (age ≥ 35 years) of Chinese ethnicity were enrolled in Taiwan (starting in 1990), and data were collected from 1990 to 2001 [16] for prospective follow-up of patients with CAD.

Among the CCCC study individuals, 90 (72 men and 18 women) with CAD were identified. CAD was defined as angiographically proven stenosis (>50%) of at least 1 major epicardial coronary artery. The non-CAD group comprised 200 subjects (96 men and 104 women) who were CCCC study individuals referred to the National Taiwan University Hospital for cardiac examinations and showed no clinical, electrocardiographic, or angiographic manifestations of CAD. All subjects underwent measurement of plasma lipid levels ≥3 times; none received lipid-lowering agents within 12 weeks before blood sampling. Cardiovascular risk factors were documented.

### Blood collection

Venous blood was collected from subjects after overnight fasting. Plasma from each sample was obtained by centrifuging at 300 × *g* at 4°C for 15 min. Fresh plasma samples were used for lipid and Apo analysis and for lipoprotein preparations. VLDL (density <1.006 kg/L) and HDL (density = 1.063–1.21 kg/L) fractions were isolated by sequential ultracentrifugation by using a Beckman 50.4 Ti Rotor Assembly (Fullerton, CA, USA) [17]. The composition of isolated lipoproteins was analyzed for TC, ApoCIII, and ApoE content.

### Assessment of lipid profile and apolipoproteins

TC and TG concentrations were measured enzymatically in an Eppendorf Epose 5060 autoanalyzer (Hamburg, Germany) by using commercial kits (Merck Chemical Co., Germany) [15]. Lipoprotein (a) [Lp(a)] concentration, regardless of the isoform, was determined by using an enzyme-linked immunosorbent assay (ELISA; Organon Teknika, MD, USA). ApoAI and ApoB concentrations were measured by using commercially available immunoturbidimetric assay kits (Sigma Chemical Co., St. Louis, MO, USA) [16]. ApoCIII and ApoE concentrations were determined by using an ELISA method developed in our laboratory [18].

### Statistical analysis

The natural logarithm of Lp(a) levels was used to normalize their right-skewed distribution. After forced entry of Apo covariables, multivariate logistic regression analysis was used to determine adjusted odds ratios (ORs) and 95% confidence intervals (CIs). Receiver operating characteristic (ROC) curve analysis was performed to compare the ability of different lipid factors to discriminate between subjects with CAD and those without CAD. All analyses were performed by using SAS, version 6.11 (SAS Institute Inc., Cary, NC, USA). A *P*-value <0.05 was considered statistically significant.

## Results

The mean age of CAD patients and non-CAD subjects was 57.4±10.9 years and 53.4±10.7 years, respectively (*P*=0.233). Mean lipid and Apo values for CAD and non-CAD groups are shown in Table 1, and these data are stratified by sex in Additional file 1. Levels of LDL-C and ApoB, both conventional markers for CAD risk, were not significantly different between the CAD and non-CAD groups. However, CAD patients had significantly higher TC and lower HDL-C and ApoAI levels than non-CAD subjects. We found that CAD patients had significantly higher HDL-ApoCIII and lower VLDL-ApoCIII levels than non-CAD subjects, but significant intergroup difference were not observed in total plasma ApoCIII, Lp(a), ApoE, HDL-ApoE, or VLDL-ApoE levels.

**Table 1:** **Lipid and apolipoprotein parameters in non**-**CAD and CAD subjects**

	**Non**-**CAD** (**N**=**200**)	**CAD** (**N**=**90**)	***P*value**
**Men**, **%**	48.0	80.0	0.01
**Age**, **yr**	53.4±10.7	57.4±10.9	0.233
**Parameters** (**mg**/**dL**±**SEM**)			
** TG**	223.2±15.8	261.9±12.4	0.07
** TC**	224.3±4.3	244.8±7.0	0.01
** HDL**-**C**	42.5±0.8	37.0±1.2	0.0003
** LDL**-**C**	165.5±4.1	174.6±6.7	0.23
** ApoAI**	130.4±2.3	77.8±5.7	<0.0001
** ApoB**	97.9±7.8	111.7±3.1	0.10
** Lp**(**a**)	1.3±0.1	1.1±0.2	0.36
** ApoCIII**	18.9±0.4	17.4±0.6	0.06
** HDL**-**ApoCIII**	9.4±0.2	12.3±0.4	<0.0001
** VLDL**-**ApoCIII**	9.9±0.3	5.5±0.4	<0.0001
** ApoE**	4.2±0.1	5.0±0.3	0.05
** VLDL**-**ApoE**	1.3±0.1	1.7±0.3	0.18
** HDL**-**ApoE**	3.0±0.1	3.4±0.2	0.07

Data are expressed as the mean ± standard error of the mean (SEM)

CAD = coronary artery disease; TG = triglyceride; TC = total cholesterol; HDL-C = high-density lipoprotein cholesterol; LDL-C = low-density lipoprotein cholesterol; Apo = apolipoprotein; Lp(a) = lipoprotein (a); HDL = high-density lipoprotein; VLDL = very-low-density lipoprotein.

Because not all CAD patients had high plasma TC or TG levels, we examined the correlation of lipid and Apo parameters with CAD by dividing all subjects into 4 groups on the basis of their lipidemic phenotypes: normolipidemic (NL), hypertriglyceridemic (HTG; TG, ≥150 mg/dL), hypercholesterolemic (HC; TC, ≥200 mg/dL), and hyperlipidemic (HLP; HTG + HC). Although plasma TG levels in the NL, HTG, and HC groups were higher in CAD patients than in non-CAD subjects, the differences were not significant. However, TG levels in the HLP group were significantly lower in CAD patients than in non-CAD patients (Table 2 and Additional file 2). Notably, plasma TC and LDL-C concentrations were not significantly different between CAD patients and non-CAD subjects in any group, except for the HLP group. The plasma ApoCIII concentration was lower in CAD patients than in non-CAD subjects in the HTG, HC, and HLP groups (Table 2). In contrast, in the NL group, CAD patients had higher levels of plasma ApoCIII than non-CAD subjects. Therefore, plasma total ApoCIII level could not be considered a unanimous marker for CAD.

**Table 2:** Correlation of lipid and apolipoprotein parameters with CAD in various lipidemic groups

	**NL**			**HTG**^a^			**HC**^b^			**HLP**^c^		
	**(n=40)**	**(n=15)**		**(n=59)**	**(n=11)**		**(n=36)**	**(n=29)**		**(n=65)**	**(n=35)**	
**CAD**	–	+		–	+		–	+		–	+	
			***P*value**			***P*value**			***P*value**			***P*value**
**TG**	78.9±17.3	101.4±26.0	NS	345.4±15.0	396.0±33.6	NS	90.4±17.5	110.2±19.8	NS	391.5±12.9	333.2±17.5	0.008
**TC**	160.6±5.8	175.8±8.7	NS	171.7±5.0	178.3±11.3	NS	275.4±5.9	261.6±6.6	NS	280.9±4.3	272.6±5.9	NS
**LDL**-**C**	103.2±6.3	119.8±9.5	NS	120.8±5.5	104.1±12.3	NS	210.1±6.4	201.6±7.2	NS	219.3±4.7	199.2±6.5	0.01
**HDL**-**C**	47.5±1.7	35.8±2.6	0.0004	38.0±1.5	26.4±3.4	0.0025	53.2±1.8	38.6±2.0	0.0001	39.5±1.3	36.2±1.8	NS
**Lp**(**a**)	2.19±0.39	4.41±0.26	0.0001	3.38±0.22	3.69±0.87	NS	4.54±0.26	3.23±0.33	0.001	4.34±0.19	4.86±0.35	NS
**ApoAI**	122±4.9	95.9±6.7	0.002	127±5.5	108±8.6	NS	138±4.5	107±5.0	0.001	135±3.4	114±4.5	0.0003
**ApoB**	75±5.3	79±7.3	NS	85.6±6.0	127±9.4	0.0002	113±4.9	114±5.5	NS	142±3.7	152±4.9	NS
**ApoCIII**	14.5±0.9	18.4±1.3	0.02	16.8±0.8	16.0±1.7	NS	21.3±0.9	17.9±1.0	0.01	22.4±0.6	16.5±0.9	0.0001
**ApoE**	3.33±0.34	2.96±0.52	NS	4.30±0.30	6.63±0.67	0.002	4.18±0.35	3.79±0.33	NS	4.98±0.26	6.13±0.37	0.01

Data are presented as mean ± standard error of the mean, and all units are mg/dL.

CAD = coronary artery disease; NL = normolipidemic; HTG = hypertriglyceridemic; HC = hypercholesterolemic; HLP = hyperlipidemic; TG = triglyceride; NS = nonsignificant; TC = total cholesterol; LDL-C = low-density lipoprotein cholesterol; HDL-C = high-density lipoprotein cholesterol; Lp(a) = lipoprotein (a); Apo = apolipoprotein.

^a^TG >150 mg/dL.

^b^TC >200 mg/dL.

^c^HC + HTG.

Plasma Lp(a) levels were significantly higher in the NL group of CAD patients than of non-CAD subjects. In addition, plasma Lp(a) levels were significantly lower in the HC group of CAD patients than of non-CAD subjects. No significant difference in plasma Lp(a) levels was observed between CAD patients and non-CAD subjects in the HTG and HLP groups (Table 2). Plasma ApoAI levels in the NL, HC, and HLP groups were significantly lower in CAD patients than in non-CAD subjects—a trend not consistent with that seen for HDL-C, the major carrier of ApoAI (Table 2). The plasma content of ApoB was similar between CAD patients and non-CAD subjects in all groups. Plasma ApoE concentration, though not statistically different between CAD and non-CAD subjects, was higher in CAD patients in the HTG and HLP groups. Thus, the presence of CAD was related to reduced ApoCIII concentration and increased ApoE concentration in subjects with high cholesterol and/or TG levels (Table 2).

Because ApoCIII and ApoE are transferable between VLDL and HDL, they may play different roles in CAD development [19]; therefore, we examined the levels of ApoCIII and ApoE associated with VLDL and HDL. The presence of CAD correlated with increased levels of VLDL-ApoE in the HLP group and increased levels of HDL-ApoE in the HTG group (Table 3 and Additional file 3). In addition, the presence of CAD was correlated with decreased VLDL-ApoCIII levels in the HTG, HC, and HLP groups and increased HDL-ApoCIII levels in the NL, HTG, and HC groups (Table 3). This resulted in an HDL-ApoCIII/VLDL-ApoCIII ratio that was significantly higher in CAD patients than in non-CAD subjects in all groups (*P*=0.002–0.0001; Table 3), with the lowest ratio value in CAD patients being 3.6 and the lowest ratio value in non-CAD subjects being 1.8 (Table 3).

**Table 3:** Correlation of VLDL- and HDL-associated ApoE and ApoCIII with CAD in various lipidemic groups

	**NL**			**HTG**^a^			**HC**^b^			**HLP**^c^		
	**(n=40)**	**(n=15)**		**(n=59)**	**(n=11)**		**(n=36)**	**(n=29)**		**(n=65)**	**(n=35)**	
	**–**	**+**		**–**	**+**		**–**	**+**		**–**	**+**	
			***P*value**			***P*value**			***P*value**			***P*value**
**VLDL**-**ApoE**	1.3±0.2	0.6±0.3	NS	1.60±0.2	2.1±0.4	NS	1.1±0.2	0.8±0.2	NS	1.1±0.1	2.0±0.2	0.002
**HDL**-**ApoE**	1.9±0.2	2.3±0.3	NS	2.7±0.2	4.4±0.4	0.001	3.1±0.2	2.8±0.2	NS	3.8±0.1	4.1±0.2	NS
**VLDL**-**ApoCIII**	6.4±0.7	4.7±1.0	NS	9.3±0.6	3.8±1.3	0.0004	14.0±0.7	5.9±0.8	0.0001	9.6±0.5	5.1±0.7	0.0001
**HDL**-**ApoCIII**	8.3±0.5	13.7±0.8	0.0001	7.5±0.4	12.1±1.0	0.0001	7.2±0.5	12.0±0.6	0.0001	12.7±0.4	11.4±0.5	NS
**HDL**-**ApoCIII to VLDL**-**ApoCIII ratio**	1.8±0.5	4.6±0.7	0.002	1.1±0.4	8.4±1.0	0.0001	0.6±0.5	3.6±0.6	0.0001	1.6±0.4	4.2±0.5	0.0001

Data are presented as mean ± standard error of the mean, and all units are mg/dL, except for the HDL-ApoCIII to VLDL-ApoCIII ratio.

VLDL = very low-density lipoprotein; HDL = high-density lipoprotein; Apo = apolipoprotein; CAD = coronary artery disease; NL = normolipidemic; HTG = hypertriglyceridemic; HC = hypercholesterolemic; HLP = hyperlipidemic; NS = nonsignificant.

^a^TG > 150 mg/dL.

^b^TC > 200 mg/dL.

^c^HC + HTG.

**Figure 1:**
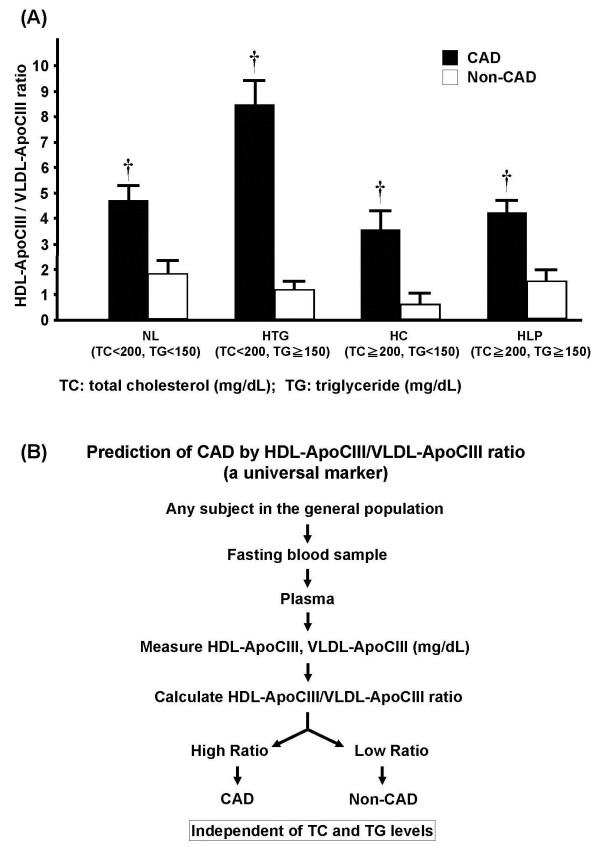
**The receiver operating characteristic** (**ROC**) **curves for various CAD predictors.** Five ratios (HDL-ApoCIII/VLDL-ApoCIII, ApoB/ApoAI, TC/HDL-C, HDL-C/LDL-C, and HDL-ApoE/VLDL-ApoE) were analyzed and compared as indicated. Area under the curve (AUC) values are shown.

To assess the association between CAD and various lipid and Apo parameters, we performed multivariate logistic regression analyses by forcing entry of HDL-ApoCIII, VLDL-ApoCIII, and other covariables that were significantly different between groups in the univariate analysis. Results of the analyses (Table 4) showed that CAD patients had a significantly higher HDL-ApoCIII and/or lower VLDL-ApoCIII concentration than non-CAD subjects in all groups. When the HDL-ApoCIII/VLDL-ApoCIII ratio was entered in the final model, it was shown to be a much stronger predictor of CAD than were the levels of TC, LDL-C, TG, or ApoB (Table 4). Receiver operating characteristic (ROC) curve analysis was performed to compare the ability of different lipid factors to discriminate between CAD and non-CAD subjects. Lipid factors compared included HDL-ApoCIII/VLDL-ApoCIII, HDL-C/LDL-C, TC/HDL-C, ApoB/ApoAI, and HDL-ApoE/VLDL-ApoE. As shown in Figure 1, HDL-C/LDL-C, TC/HDL-C, ApoB/ApoAI, and HDL-ApoCIII/VLDL-ApoCIII were all able to discriminate between subjects with CAD and those without CAD, whereas HDL-ApoE/VLDL-ApoE could not. Among the four significant CAD predictors identified, HDL-ApoCIII/VLDL-ApoCIII had the strongest ability to predict CAD occurrence (c-statistic, 0.82 [95% confidence interval, 0.76-0.88]; overall *P* <0.001).

**Table 4:** Odds ratios and 95% confidence intervals for predicting CAD in various lipidemic groups and in all subjects

	**Odds Ratio**	**95%CI**	***P*value**
**NL Group**^**a**^			
HDL-ApoCIII	1.49	1.12–1.99	0.006
VLDL-ApoCIII	0.74	0.53–1.01	0.06
HDL-ApoCIII/ VLDL-ApoCIII	1.85	1.14–2.89	0.01
Men	2.76	1.57–3.48	0.005
Women	1.65	1.06–2.33	0.02
ApoAI	0.62	0.46–0.84	0.002
**HTG Group**^**a**^			
HDL-ApoCIII	2.00	1.05–3.80	0.04
VLDL-ApoCIII	0.79	0.45–1.41	0.43
HDL-ApoCIII/ VLDL-ApoCIII	2.52	0.76–5.98	0.02
Men	3.17	1.14–3.94	0.005
Women	1.55	1.04–1.89	0.03
**HC Group**^**a**^			
HDL-ApoCIII	1.56	1.18–2.05	0.002
VLDL-ApoCIII	0.74	0.61–0.90	0.003
HDL-ApoCIII/ VLDL-ApoCIII	1.78	1.23–2.67	0.003
Men	1.96	1.26–2.67	0.005
Women	1.23	1.06–1.89	0.01
ApoAI	0.72	0.55–0.93	0.01
**HLP Group**^**a**^			
HDL-ApoCIII	0.90	0.64–1.28	0.57
VLDL-ApoCIII	0.66	0.54–0.81	<0.0001
HDL-ApoCIII/ VLDL-ApoCIII	1.57	1.01–2.24	0.01
Men	1.80	1.26–2.72	0.004
Women	1.25	0.94–1.88	0.02
TG	0.96	0.94–0.99	0.005
TC	1.16	1.04–1.30	0.006
LDL-C	0.83	0.73–0.94	0.004
ApoAI	0.21	0.07–0.68	0.009
**All Subjects**, **Final Model**^**b**^			
HDL-ApoCIII/ VLDL-ApoCIII	2.04	1.46–2.84	<0.0001
Female sex	0.26	0.10–0.66	0.0043
Age	1.06	1.02–1.10	0.0063
TC	1.04	1.01–1.06	0.002
TG	0.99	0.99–1.00	<0.0001
LDL-C	0.99	0.98–1.00	0.01
HDL-C	0.95	0.90–1.00	0.03
ApoB	1.46	1.25–1.70	<0.0001
ApoAI	0.66	0.55–0.80	<0.0001

CAD = coronary artery disease; CI = confidence interval; NL = normolipidemic; HDL = high-density lipoprotein; VLDL = very-low-density lipoprotein; Apo = apolipoprotein; HTG = hypertriglyceridemic; HC = hypercholesterolemic; HLP = hyperlipidemic; TG = triglyceride; TC = total cholesterol; LDL-C = low-density lipoprotein cholesterol.

^a^The following co-variables were entered in the multivariate regression model: TG, TC, ApoAI, ApoB, ApoCIII, ApoE, LDL-C, HDL-C, HDL-ApoE, VLDL-ApoE, and forced entry of HDL-ApoCIII and VLDL-ApoCIII.

^b^The following co-variables were entered in the multivariate regression model: TG, TC, ApoAI, ApoB, Lp(a), ApoCIII, ApoE, LDL-C, HDL-C, HDL-ApoE, VLDL-ApoE and HDL-ApoCIII/VLDL-ApoCIII ratio.

## Discussion

**Figure 2:**
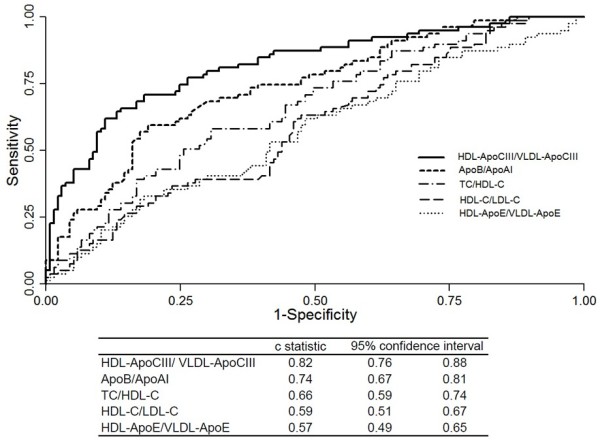
(**a**) **Graph showing the ratio of high**-**density lipoprotein** (**HDL**)–**apolipoprotein** (**Apo**) **CIII to very**-**low**-**density lipoprotein** (**VLDL**)–**ApoCIII in study groups categorized by total cholesterol** (**TC**) **and triglyceride** (**TG**) **levels.** Each study group was further divided according to the presence of coronary artery disease (CAD, black bar; non-CAD, white bar). Values are presented as the mean ± standard error of the mean; †*P*<0.01 for CAD vs. non-CAD. NL, normolipidemic; HTG, hypertriglyceridemic; HC, hypercholesterolemic; HLP, hyperlipidemic. (b) Flow diagram proposing the use of the HDL-ApoCIII/VLDL-ApoCIII ratio as a predictor for CAD in the general population.

In our study, HDL-ApoCIII was positively associated with the presence of CAD, and VLDL-ApoCIII was inversely associated with the presence of CAD. We further identified the HDL-ApoCIII/VLDL-ApoCIII ratio to be a predictor of CAD. Adjustments for age and sex increased the ratio’s predictive potential. To our knowledge, we are the first to report that the ApoCIII/VLDL-ApoCIII ratio is a unanimous predictor of CAD, irrespective of lipidemic phenotype. We demonstrate that, regardless of TG and TC levels, a high HDL-ApoCIII/VLDL-ApoCIII ratio suggests the presence of CAD, and a low HDL-ApoCIII/VLDL-ApoCIII ratio suggests the absence of CAD (Figure 2). Compared to other lipid factors such as HDL-C/LDL-C, TC/HDL-C, and ApoB/ApoAI, the HDL-ApoCIII/VLDL-ApoCIII ratio has the strongest ability to predict CAD occurrence (Figure 1).

The Cholesterol and Recurrent Events trial showed that plasma VLDL-ApoCIII concentration and hypertriglyceridemia are positively associated with the incidence of cardiovascular disease [20]. In diabetic patients, ApoCIII level in ApoB lipoprotein is an independent predictor of risk for coronary events [21]. Most plasma ApoCIII is found on the surface of VLDL and LDL; a higher percentage (approximately 30%-70%) of VLDL than HDL contains ApoCIII [22]. Excessive ApoCIII delays lipolysis of VLDL and inhibits its uptake and clearance from plasma by normal high-affinity receptors on hepatocytes [23]. Therefore, our findings suggest a massive transfer of ApoCIII from VLDL to HDL in CAD patients and indicate a link between compositional changes of ApoCIII in lipoproteins and CAD [24]. The transfer of ApoCIII is accompanied by tremendous compositional changes that may contribute to the progression of atherosclerosis. In addition, increased ApoCIII levels in HDL may attenuate the anti-atherogenic effects of ApoE, thereby accelerating coronary atherosclerosis [25].

Although LDL-C and HDL-C are the recommended lipid variables in the international guidelines for the treatment of CAD [1], we have found that the HDL-ApoCIII/VLDL-ApoCIII ratio is a better predictor of CAD than LDL-C or ApoB levels in individuals with normal TC and TG levels. This finding implies that LDL-C or its components such as ApoB may not suffice as predictors of CAD. Moreover, this ratio may be a predictor of CAD in diabetic individuals in whom atherogenicity is mainly attributed to VLDL, TG-rich lipoproteins, and abnormal HDL particles [26].

In our study, the HDL-ApoCIII/VLDL-ApoCIII ratio was the strongest predictor of CAD in the general population and represents an accurate measure of atherogenic HDL-ApoCIII and VLDL-ApoCIII particles rather than a measure of lipids levels per particle. This ratio may help to identify individuals at risk of angiographically significant CAD, irrespective of lipid concentrations, and it may also be useful in clinical practice for routinely assessing CAD risk in the general population and for making decisions about diet and pharmacologic treatment. Importantly, the assays for analyzing the HDL-ApoCIII/VLDL-ApoCIII ratio are automated, accurate, and inexpensive, making this parameter applicable for routine use in the clinical setting.

Notably, numerous results of CCCC-based studies are in accordance with findings in other ethnic populations [15,16,27]. The CCCC population has lower HDL-C and ApoAI concentrations than other international populations, such as the Framingham and the third National Health and Nutrition Examination Survey populations, but the mean ApoB level is approximately the same among these populations [28]. Because most of the subjects included in this study had not been treated for CAD at the time of referral, the bias from pharmacotherapy is minimal [15]. Furthermore, bias in the recruitment of individuals was negligible because patients in the CCCC were unaware that their apolipoprotein levels would be measured. Therefore, we believe our conclusions may be truly representative of a large population.

### Study limitations

Because of the lack of longitudinal assessment of biochemical markers in our study, we could not account for the effects of serial changes in the HDL-ApoCIII/VLDL-ApoCIII ratio that may be associated with the incidence of cardiovascular events. In addition, we did not obtain complete information regarding the study subjects’ medical therapy, preventing us from analyzing the influence of medications on ApoCIII concentration. Finally, the lipid cutoff value defined in our study has not been tested in an independent data set or prospectively validated.

## Conclusions

The HDL-ApoCIII/VLDL-ApoCIII ratio is a better predictor of CAD risk than are the conventional lipid factors, even though levels of plasma lipids ApoAI and ApoB remain important predictors for CAD. In addition to its potential clinical importance, high levels of ApoCIII-containing HDL may represent a class of dysfunctional HDL—a topic that has gained much attention in atherogenesis research [29,30].

## Abbreviations

Apo: Apolipoprotein; AUC: Area under the curve; CAD: Coronary artery disease; CCCC: Chin-Shan Community Cardiovascular Cohort; CI: Confidence interval; HC: Hypercholesterolemic; HDL-C: High-density lipoprotein cholesterol; HLP: Hyperlipidemic; HTG: Hypertriglyceridemic; LDL-C: Low-density lipoprotein cholesterol; Lp(a): Lipoprotein (a); NL: Normolipidemic; OR: Odds ratio; ROC: Receiver operating characteristic; TC: Total cholesterol; TG: Triglyceride; VLDL: Very-low-density lipoprotein.

## Competing interests

The authors declare that they have no competing interests.

## Authors’ contributions

All authors made substantial contributions to the conception and design of the study and manuscript, contributing to the drafting of the article or revising it critically for important contents, and all gave final approval of the version to be published.

## Supplementary Material

Additional file 1 Lipid and apolipoprotein parameters (stratified by sex) in non-CAD and CAD subjects. Click here for file

Additional file 2 Correlation of lipid and apolipoprotein parameters with CAD (stratified by sex) in various lipidemic groups. Click here for file

Additional file 3 Correlation of VLDL- and HDL-associated ApoE and ApoCIII with CAD (stratified by sex) in various lipidemic groups. Click here for file
