# Supplementary material for: Identification of the HDL-ApoCIII to VLDL-ApoCIII ratio as a predictor of coronary artery disease in the general population: The Chin-Shan Community Cardiovascular Cohort (CCCC) study in Taiwan
Source: Lipids Health Dis. 2012 Nov 23;11:162. doi: 10.1186/1476-511X-11-162 (PMC3543287; doi:10.1186/1476-511X-11-162)
Supplement: Additional file 1 — Lipid and apolipoprotein parameters (stratified by sex) in non-CAD and CAD subjects. [file 1476-511X-11-162-S1.doc]

**Additional File 1** Lipid and apolipoprotein parameters (stratified by sex) in non-CAD and CAD subjects

|  | **Women (n=122)** | | | | | |  | **Men (n=168)** | | | | | |
| --- | --- | --- | --- | --- | --- | --- | --- | --- | --- | --- | --- | --- | --- |
|  | Non-CAD (n=104) | | | CAD (n=18) | | |  | Non-CAD (n=96) | | | CAD (n=72) | | |
| **Age, yr** | 53.7 | ± | 9.7 | 60.8 | ± | 8.2^§^ |  | 53.0 | ± | 11.8 | 56.5 | ± | 11.4^§^ |
| **TG** | 254.6 | ± | 18.3 | 261.6 | ± | 32.5 |  | 269.9 | ± | 16.8 | 249.5 | ± | 40.0 |
| **TC** | 229.2 | ± | 6.1 | 266.1 | ± | 20.2^§^ |  | 219.1 | ± | 6.1 | 239.6 | ± | 8.2^§^ |
| **HDL-C** | 43.6 | ± | 1.2 | 38.1 | ± | 2.9^¶^ |  | 41.4 | ± | 1.2 | 36.8 | ± | 1.4^¶^ |
| **LDL-C** | 171.7 | ± | 5.7 | 190.9 | ± | 14.0 |  | 158.6 | ± | 5.6 | 170.8 | ± | 7.5 |
| **ApoAI** | 132.2 | ± | 2.8 | 105.5 | ± | 8.8^¶^ |  | 128.5 | ± | 3.1 | 71.5 | ± | 13.2^¶^ |
| **ApoB** | 112.2 | ± | 3.8 | 130.0 | ± | 12.1 |  | 111.1 | ± | 4.3 | 90.0 | ± | 18.1 |
| **Lp(a)** | 1.3 | ± | 0.4 | 1.8 | ± | 0.1^§^ |  | 1.3 | ± | 0.2 | 0.5 | ± | 0.2 |
| **ApoCIII** | 18.0 | ± | 0.7 | 19.3 | ± | 1.2 |  | 17.2 | ± | 0.8 | 16.5 | ± | 1.6 |
| **HDL-ApoCIII** | 9.5 | ± | 0.3 | 13.1 | ± | 1.1^¶^ |  | 9.3 | ± | 0.4 | 12.1 | ± | 1.1^¶^ |
| **VLDL-ApoCIII** | 9.4 | ± | 0.5 | 7.0 | ± | 1.2^¶^ |  | 10.5 | ± | 0.5 | 5.1 | ± | 1.1^¶^ |
| **ApoE** | 4.4 | ± | 0.2 | 5.9 | ± | 1.0 |  | 4.0 | ± | 0.3 | 4.6 | ± | 0.7 |
| **VLDL-ApoE** | 1.3 | ± | 0.1 | 2.0 | ± | 0.5 |  | 1.4 | ± | 0.1 | 1.7 | ± | 0.7 |
| **HDL-ApoE** | 3.1 | ± | 0.2 | 4.4 | ± | 0.5 |  | 2.9 | ± | 0.1 | 3.2 | ± | 0.4 |

Data are expressed as the mean ± standard error of the mean, and all units are mg/dL except for age.

^§^*P*<0.05 vs. non-CAD group

^¶^*P*<0.01 vs. non-CAD group

CAD = coronary artery disease; TG = triglyceride; TC = total cholesterol; HDL-C = high-density lipoprotein cholesterol; LDL-C = low-density lipoprotein cholesterol; Apo = apolipoprotein; Lp(a) = lipoprotein(a); HDL = high-density lipoprotein; VLDL = very-low-density lipoprotein.
