# Supplementary material for: Identification of the HDL-ApoCIII to VLDL-ApoCIII ratio as a predictor of coronary artery disease in the general population: The Chin-Shan Community Cardiovascular Cohort (CCCC) study in Taiwan
Source: Lipids Health Dis. 2012 Nov 23;11:162. doi: 10.1186/1476-511X-11-162 (PMC3543287; doi:10.1186/1476-511X-11-162)
Supplement: Additional file 2 — Correlation of lipid and apolipoprotein parameters with CAD (stratified by sex) in various lipidemic groups. [file 1476-511X-11-162-S2.doc]

**Additional File 2** Correlation of lipid and apolipoprotein parameters with CAD (stratified by sex) in various lipidemic groups

Men

|  | **NL** | | | | | |  | **HTG^a^** | | | | | |  | **HC^b^** | | | | | |  | **HLP^c^** | | | | | |
| --- | --- | --- | --- | --- | --- | --- | --- | --- | --- | --- | --- | --- | --- | --- | --- | --- | --- | --- | --- | --- | --- | --- | --- | --- | --- | --- | --- |
|  | Non-CAD (n=17) | | | CAD (n=15) | | |  | Non-CAD (n=35) | | | CAD (n=7) | | |  | Non-CAD (n=13) | | | CAD (n=25) | | |  | Non-CAD (n=31) | | | CAD (n=25) | | |
| **TG** | 70.8 | ± | 4.8 | 101.5 | ± | 8.1 |  | 329.9 | ± | 15.4 | 405.6 | ± | 11.0 |  | 90.3 | ± | 6.3 | 108.0 | ± | 5.8 |  | 386.5 | ± | 26.4 | 436.1 | ± | 101.1^§^ |
| **TC** | 161.5 | ± | 5.5 | 175.8 | ± | 4.1 |  | 171.6 | ± | 3.6 | 176.1 | ± | 5.1 |  | 267.5 | ± | 3.8 | 261.7 | ± | 15.4 |  | 284.2 | ± | 5.3 | 273.6 | ± | 10.3 |
| **LDL-C** | 103.8 | ± | 4.8 | 119.8 | ± | 4.0 |  | 122.0 | ± | 3.6 | 102.6 | ± | 6.4 |  | 206.3 | ± | 4.6 | 200.1 | ± | 15.4 |  | 217.4 | ± | 7.4 | 191.3 | ± | 7.6^§^ |
| **HDL-C** | 47.2 | ± | 1.6 | 35.9 | ± | 2.2^¶^ |  | 37.1 | ± | 1.8 | 26.9 | ± | 1.2^¶^ |  | 48.4 | ± | 3.3 | 39.9 | ± | 2.2^§^ |  | 40.0 | ± | 2.0 | 36.9 | ± | 2.7^§^ |
| **Lp(a)** | 1.4 | ± | 0.3 | 2.0 | ± | 0.6^¶^ |  | 6.7 | ± | 1.0 | 5.5 | ± | 4.0^¶^ |  | 2.6 | ± | 0.6 | 4.9 | ± | 1.6^¶^ |  | 1.3 | ± | 0.2 | 0.7 | ± | 0.3^¶^ |
| **ApoAI** | 124.4 | ± | 25.3 | 100 | ± | 5.0^¶^ |  | 118.6 | ± | 5.5 | 111.0 | ± | 6.4 |  | 130.0 | ± | 9.7 | 51.2 | ± | 10.1^¶^ |  | 135.0 | ± | 5.5 | 117.7 | ± | 6.0^§^ |
| **ApoB** | 77.1 | ± | 4.3 | 80.2 | ± | 5.1^¶^ |  | 82.0 | ± | 3.1 | 130.0 | ± | 8.3^¶^ |  | 116 | ± | 4.7 | 60.7 | ± | 13.1^¶^ |  | 147.0 | ± | 47.3 | 157.2 | ± | 10.0 |
| **ApoCIII** | 12.9 | ± | 2.1 | 18.5 | ± | 2.1 |  | 15.3 | ± | 0.9 | 15.7 | ± | 2.1 |  | 19.1 | ± | 1.6 | 16.7 | ± | 1.7 |  | 21.0 | ± | 1.4 | 15.4 | ± | 0.8^§^ |
| **ApoE** | 3.4 | ± | 0.4 | 3.0 | ± | 0.2 |  | 4.1 | ± | 0.4 | 6.7 | ± | 0.7^¶^ |  | 3.5 | ± | 0.2 | 3.7 | ± | 0.3 |  | 4.5 | ± | 0.3 | 6.0 | ± | 0.9 |

Women

|  | **NL** | | | |  | **HTG^a^** | | | | | |  | **HC^b^** | | | | | |  | **HLP^c^** | | | | | |
| --- | --- | --- | --- | --- | --- | --- | --- | --- | --- | --- | --- | --- | --- | --- | --- | --- | --- | --- | --- | --- | --- | --- | --- | --- | --- |
|  | Non-CAD (n=23) | | | CAD (n=1) |  | Non-CAD (n=24) | | | CAD (n=2) | | |  | Non-CAD (n=23) | | | CAD (n=5) | | |  | Non-CAD (n=34) | | | CAD (n=10) | | |
| **TG** | 80.7 | ± | 6.1 | 149.0 |  | 366.8 | ± | 25.0 | 362.5 | ± | 10.5 |  | 91.5 | ± | 6.6 | 110.6 | ± | 10.9 |  | 403.4 | ± | 26.4 | 328.2 | ± | 38.9^§^ |
| **TC** | 162.6 | ± | 4.1 | 198.0 |  | 173.0 | ± | 37.2 | 186.0 | ± | 3.0 |  | 279.0 | ± | 5.3 | 271.0 | ± | 10.9 |  | 280.3 | ± | 5.0 | 286.4 | ± | 14.8 |
| **LDL-C** | 105.4 | ± | 3.5 | 130.0 |  | 122.7 | ± | 4.9 | 109.5 | ± | 5.5 |  | 214.5 | ± | 5.9 | 197.2 | ± | 18.7 |  | 220.9 | ± | 4.6 | 212.3 | ± | 18.7 |
| **HDL-C** | 47.7 | ± | 2.0 | 49.0 |  | 36.6 | ± | 2.9 | 25.0 | ± | 1.0^¶^ |  | 52.9 | ± | 2.8 | 49.8 | ± | 6.3 |  | 39.4 | ± | 1.3^§^ | 33.7 | ± | 2.0^§^ |
| **Lp(a)** | 1.6 | ± | 0.3 | 0.5 |  | 0.3 | ± | 0.1 | 1.1 | ± | 1.1^¶^ |  | 2.1 | ± | 0.3 | 1.0 | ± | 0.6^¶^ |  | 1.3 | ± | 0.2 | 2.5 | ± | 0.8^¶^ |
| **ApoAI** | 122.0 | ± | 4.2 | 120 |  | 132.0 | ± | 3.7 | 103.0 | ± | 10.0^¶^ |  | 137.6 | ± | 7.9 | 101.0 | ± | 25.1^¶^ |  | 136.0 | ± | 4.2^§^ | 117.0 | ± | 2.8^§^ |
| **ApoB** | 74.0 | ± | 4.9 | 80 |  | 98.9 | ± | 3.5 | 122.0 | ± | 5.1 |  | 115.0 | ± | 5.0 | 100.1 | ± | 24.7 |  | 138.0 | ± | 6.1^§^ | 159.0 | ± | 7.2^§^ |
| **ApoCIII** | 12.0 | ± | 1.2 | 24.1 |  | 15.5 | ± | 1.4 | 17.1 | ± | 2.5 |  | 21.8 | ± | 1.2 | 19.6 | ± | 2.6 |  | 21.1 | ± | 1.1 | 19.2 | ± | 1.9 |
| **ApoE** | 3.3 | ± | 0.2 | 4.1 |  | 4.4 | ± | 0.6 | 6.6 | ± | 2.4 |  | 4.4 | ± | 0.2 | 4.5 | ± | 1.1 |  | 5.0 | ± | 0.3 | 6.6 | ± | 1.4 |

Data are presented as mean ± standard error of the mean, and all units are mg/dL.

^§^*P*<0.05 vs. non-CAD group

^¶^*P*<0.01 vs. non-CAD group

CAD = coronary artery disease; NL = normolipidemic; HTG = hypertriglyceridemic; HC = hypercholesterolemic; HLP = hyperlipidemic; TG = triglyceride; NS = nonsignificant; TC = total cholesterol; LDL-C = low-density lipoprotein cholesterol; HDL-C = high-density lipoprotein cholesterol; Lp(a) = lipoprotein (a); Apo = apolipoprotein.

^a^TG>150 mg/dL

^b^TC>200 mg/dL

^c^HC+HTG
