# Supplementary material for: Identification of the HDL-ApoCIII to VLDL-ApoCIII ratio as a predictor of coronary artery disease in the general population: The Chin-Shan Community Cardiovascular Cohort (CCCC) study in Taiwan
Source: Lipids Health Dis. 2012 Nov 23;11:162. doi: 10.1186/1476-511X-11-162 (PMC3543287; doi:10.1186/1476-511X-11-162)
Supplement: Additional file 3 — Correlation of VLDL- and HDL-associated ApoE and ApoCIII with CAD (stratified by sex) in various lipidemic groups. [file 1476-511X-11-162-S3.doc]

## Additional File 3 Correlation of VLDL- and HDL-associated ApoE and ApoCIII with CAD (stratified by sex) in various lipidemic groups

Men

|  | **NL** | | | | | |  | **HTG^a^** | | | | | |  | **HC^b^** | | | | | |  | **HLP^c^** | | | | | |
| --- | --- | --- | --- | --- | --- | --- | --- | --- | --- | --- | --- | --- | --- | --- | --- | --- | --- | --- | --- | --- | --- | --- | --- | --- | --- | --- | --- |
|  | Non-CAD (n=17) | | | CAD (n=15) | | |  | Non-CAD (n=35) | | | CAD (n=7) | | |  | Non-CAD (n=13) | | | CAD (n=25) | | |  | Non-CAD (n=31) | | | CAD (n=25) | | |
| **VLDL-ApoE** | 1.5 | ± | 0.3 | 0.6 | ± | 0.2^§^ |  | 1.6 | ± | 0.3 | 2.4 | ± | 0.6^§^ |  | 1.3 | ± | 0.3 | 1.2 | ± | 0.3 |  | 1.3 | ± | 0.2 | 2.8 | ± | 0.9^§^ |
| **HDL-ApoE** | 2.0 | ± | 0.2 | 2.3 | ± | 0.3 |  | 2.7 | ± | 0.2^¶^ | 4.2 | ± | 0.5^¶^ |  | 2.4 | ± | 0.3 | 2.8 | ± | 0.4 |  | 3.8 | ± | 0.2 | 3.9 | ± | 0.4 |
| **VLDL-ApoCIII** | 9.5 | ± | 1.4 | 4.7 | ± | 1.0^¶^ |  | 9.5 | ± | 0.7 | 3.0 | ± | 1.3^¶^ |  | 13.4 | ± | 1.1 | 6.3 | ± | 1.1^¶^ |  | 10.9 | ± | 0.8 | 4.9 | ± | 0.8^¶^ |
| **HDL-ApoCIII** | 8.5 | ± | 0.6 | 13.8 | ± | 1.5^§^ |  | 7.3 | ± | 0.4 | 12.6 | ± | 1.1^¶^ |  | 7.2 | ± | 0.8 | 11.9 | ± | 0.8^¶^ |  | 12.8 | ± | 0.6 | 11.2 | ± | 0.7 |
| **HDL-ApoCIII to**  **VLDL-ApoCIII ratio** | 1.3 | ± | 0.2 | 4.6 | ± | 0.9^¶^ |  | 1.2 | ± | 0.2 | 10.3 | ± | 3.4^¶^ |  | 0.6 | ± | 0.1 | 3.1 | ± | 0.5^¶^ |  | 1.3 | ± | 0.1 | 4.9 | ± | 1.1^¶^ |

**Women**

|  | **NL** | | | |  | **HTG^a^** | | | | | |  | **HC^b^** | | | | | |  | **HLP^c^** | | | | | |
| --- | --- | --- | --- | --- | --- | --- | --- | --- | --- | --- | --- | --- | --- | --- | --- | --- | --- | --- | --- | --- | --- | --- | --- | --- | --- |
|  | Non-CAD (n=23) | | | CAD (n=1) |  | Non-CAD (n=24) | | | CAD (n=2) | | |  | Non-CAD (n=23) | | | CAD (n=5) | | |  | Non-CAD (n=34) | | | CAD (n=10) | | |
| **VLDL-ApoE** | 1.3 | ± | 0.2 | 1.1 |  | 1.6 | ± | 0.2 | 1.3 | ± | 0.5 |  | 1.1 | ± | 0.1 | 1.3 | ± | 0.3 |  | 1.1 | ± | 0.1^§^ | 2.7 | ± | 0.9^§^ |
| **HDL-ApoE** | 2.0 | ± | 0.1 | 3.0 |  | 2.9 | ± | 0.3 | 5.3 | ± | 2.8 |  | 3.4 | ± | 0.2 | 3.3 | ± | 0.1 |  | 3.9 | ± | 0.2 | 4.7 | ± | 0.9 |
| **VLDL-ApoCIII** | 5.3 | ± | 0.5 | 3.9 |  | 9.0 | ± | 0.7 | 6.8 | ± | 0.5^§^ |  | 14.4 | ± | 1.2 | 7.4 | ± | 3.1^§^ |  | 9.0 | ± | 0.6 | 7.1 | ± | 1.5^§^ |
| **HDL-ApoCIII** | 8.3 | ± | 0.5 | 20.2 |  | 7.9 | ± | 0.4 | 10.3 | ± | 2.0^§^ |  | 7.3 | ± | 0.3 | 15.2 | ± | 2.6^¶^ |  | 12.6 | ± | 0.5 | 12.1 | ± | 1.1 |
| **HDL-ApoCIII to**  **VLDL-ApoCIII ratio** | 2.0 | ± | 0.2 | 5.2 |  | 1.0 | ± | 0.1 | 1.5 | ± | 0.1^§^ |  | 0.6 | ± | 0.1 | 6.0 | ± | 3.1^¶^ |  | 1.9 | ± | 0.4 | 2.5 | ± | 0.5^§^ |

Data are presented as mean ± standard error of the mean, and all units are mg/dL, except for the HDL-ApoCIII to VLDL-ApoCIII ratio.

^§^*P*<0.05 vs. non-CAD group

^¶^*P*<0.01 vs. non-CAD group

VLDL = very low-density lipoprotein; HDL = high-density lipoprotein; Apo = apolipoprotein; CAD = coronary artery disease; NL = normolipidemic; HTG = hypertriglyceridemic; HC = hypercholesterolemic; HLP = hyperlipidemic; NS = nonsignificant.

^a^TG>150 mg/dL

^b^TC>200 mg/dL

^c^HC+HTG
